# Risk factors for pulmonary complications after colorectal cancer surgery: a Japanese multicenter study

**DOI:** 10.1007/s00384-024-04652-5

**Published:** 2024-05-23

**Authors:** Tetsuro Tominaga, Takashi Nonaka, Yuma Takamura, Kaido Oishi, Shintaro Hashimoto, Toshio Shiraishi, Keisuke Noda, Rika Ono, Mitsutoshi Ishii, Makoto Hisanaga, Hiroaki Takeshita, Hidetoshi Fukuoka, Shosaburo Oyama, Kazuhide Ishimaru, Masaki Kunizaki, Terumitsu Sawai, Keitaro Matsumoto

**Affiliations:** 1 Department of Surgical Oncology, Nagasaki University Graduate School of Biomedical Science, 1-7-1 Sakamoto, Nagasaki, 852-8501 Japan; 2 Department of Surgery, Sasebo City General Hospital, 9-3 Hirasemachi, Nagasaki, 857-8511 Japan; 3 Department of Surgery, National Hospital Organization Nagasaki Medical Center, 1-1001-1, Omura, Nagasaki 856-8562 Japan; 4 Department of Surgery, Isahaya General Hospital, 24-1, Isahaya, Nagasaki 854-8501 Japan; 5 Department of Surgery, Ureshino Medical Center, 4279-3 Ko, Ureshinomachi, Oaza, Shimojuku, Ureshino, Saga, 843-0393 Japan; 6 Department of Surgery, Saiseikai Nagasaki Hospital, 2-5-1 Katafuchi, Nagasaki, 850-0003 Japan; 7 Department of Surgery, Saseno Chuo Hospital, 15 Yamatocho, Sasebo, 857-1195 Japan

**Keywords:** Colorectal cancer, High age, Male, Pulmonary complication

## Abstract

**Purpose:**

Pulmonary complications (PC) are a serious condition with a 20% mortality rate. However, few reports have examined risk factors for PC after colorectal surgery. This study investigated the frequency, characteristics, and risk factors for PC after colorectal cancer surgery.

**Methods:**

Between January 2016 and December 2022, we retrospectively reviewed 3979 consecutive patients who underwent colorectal cancer surgery in seven participating hospitals. Patients were divided into patients who experienced PC (PC group, *n* = 54) and patients who did not (non-PC group, *n* = 3925). Clinical and pathological features were compared between groups.

**Results:**

Fifty-four patients (1.5%) developed PC, of whom 2 patients (3.7%) died due to PC. Age was greater (80 years vs 71 years; *p* < 0.001), frequency of chronic obstructive pulmonary distress was greater (9.3% vs 3.2%; *p* = 0.029), performance status was poorer (*p* < 0.001), the proportion of underweight was higher (42.6% vs 13.4%, *p* < 0.001), frequency of open surgery was greater (24.1% vs 9.3%; *p* < 0.001), and blood loss was greater (40 mL vs 22 mL; *p* < 0.001) in the PC group. Multivariate analysis revealed male sex (odds ratio (OR) 2.165, 95% confidence interval (CI) 1.176–3.986; *p* = 0.013), greater age (OR 3.180, 95%CI 1.798–5.624; *p* < 0.001), underweight (OR 3.961, 95%CI 2.210–7.100; *p* < 0.001), and poorer ASA-PS (OR 3.828, 95%CI 2.144–6.834; *p* < 0.001) as independent predictors of PC.

**Conclusion:**

Our study revealed male sex, greater age, underweight, and poorer ASA-PS as factors associated with development of PC, and suggested that pre- and postoperative rehabilitation and pneumonia control measures should be implemented for patients at high risk of PC.

**Supplementary Information:**

The online version contains supplementary material available at 10.1007/s00384-024-04652-5.

## Introduction

Pulmonary complications (PC) are the second most common serious postoperative complications of general surgery and represent a serious condition with a 30-day mortality rate of 20% [1]. Postoperative PC also have secondary effects, such as prolonged hospitalization and associated increases in medical costs [2, 3]. In major abdominal surgery, the incidence is reported as 3–30% [1, 4, 5].

In recent years, laparoscopic surgery has become more popular for the treatment of colorectal cancer, and good short-term postoperative outcomes have been reported in various weaning studies [6]. Laparoscopic surgery reportedly does not increase the risk of PC compared to open surgery, which is attributable to reduced surgical invasiveness, reduced pain, accelerated rehabilitation, and early recovery of organ function [1]. On the other hand, laparoscopic surgery increases the risk of hypoxemia due to decreased functional residual lung capacity, deteriorated hemodynamics, and decreased lung compliance due to increased abdominal pressure during insufflation and the steeply inclined Trendelenburg position during surgery [7–9].

Most studies on risk factors for postoperative respiratory complications have examined surgeries for the lungs, esophagus, and upper abdomen, with limited studies investigating the lower gastrointestinal tract [10–13].

The present study used a Japanese multicenter database to investigate the frequency, characteristics, and risk factors for PC following surgery for colorectal cancer.

## Materials and methods

We retrospectively reviewed 3979 consecutive patients who underwent colorectal cancer surgery in seven participating hospitals (Nagasaki University Hospital, Nagasaki Medical Center, Sasebo City General Hospital, Isahaya General Hospital, Ureshino Medical Center, Saiseikai Nagasaki Hospital, and Sasebo Chuo Hospital) between January 2016 and December 2022. Patients with only stoma construction, emergency surgery due to colon obstruction or perforation, and with recurrent tumor were excluded. The study protocol was reviewed and approved by the clinical research review boards of the participating hospitals (approval no. 16062715–5). Our study adhered to STROBE guideline.

Patients were divided into two groups: patients who experienced PC (PC group, *n* = 54) and patients who did not experience PC (non-PC group, *n* = 3925). PC were defined as respiratory failure, respiratory infection, pleural effusion, atelectasis, bronchospasm, pneumothorax, aspiration pneumonitis, pneumonia, acute respiratory distress syndrome, or pulmonary embolus.

Clinical features were compared between groups. The following data were collected: sex, age, comorbidities, preoperative treatment, American Society of Anesthesiologists performance status (ASA-PS), body mass index (BMI), clinical tumor/node/metastasis (TNM) status, and tumor location. The surgical and pathological data collected included surgical approach, operation time, blood loss, and postoperative hospital stay. Postoperative complications were defined as complications that occurred within 30 days after the primary surgery. Preoperative treatment were included preoperative chemotherapy for colon cancer and chemoradiation therapy for rectal cancer. Preoperative chemotherapy was considered for treatment of colon cancer patients with clinical T4 and/or N2. Preoperative chemoradiation was considered for treatment of rectal cancer patients with suspicious resection margin positive. The overall decision of preoperative treatment was made by the attending physician depending on the patient’s compliance, age, and the facility’s radiation therapy environment. In facilities where radiotherapy was not available, patients were treated with chemotherapy alone.

Statistical analysis was performed using JMP software (SAS Institute Inc., Cary, NC, USA). Data are presented as median and range. Differences in categorical variables were compared using the chi-squared test or Fisher’s exact test. Differences in continuous variables were analyzed using the Mann–Whitney *U* test. Multivariate analysis using logistic regression analysis was used to identify risk factors for PC. Variables with a value of *p* < 0.05 in univariate analyses were included in the multivariate analysis. All values of *p* < 0.05 were considered significant.

## Results

Table 1 lists the details of PC. Overall, 54 patients (1.5%) experienced PC, among whom 2 patients (3.7%) died due to PC. PC with Clavien-Dindo classification (CD) grade 2 occurred in 45 patients, comprising pneumonia (*n* = 37), aspiration pneumonia (*n* = 5), and atelectasis (*n* = 3). Five of these patients also had complications of ileus and six had complications of delirium. PC with CD grade 3 occurred in 3 patients, comprising pneumonia (*n* = 2) and aspiration pneumonia (*n* = 1). Two patients had complications of ileus. PC with CD grade 4 occurred 4 patients, comprising pneumonia (*n* = 1), aspiration pneumonia (*n* = 2), and respiratory failure (*n* = 1). One of these patients showed complications of ileus. PC with CD grade 5 occurred in 2 patients, comprising aspiration pneumonia (*n* = 1) and respiratory failure (*n* = 1).

**Table 1:** Details of pulmonary complications

	*n*	Other complications	*n*
CD2	45		
Pneumonia	37	Ileus	5
		Delirium	6
Aspiration pneumonia	5	Ileus	2
		Delirium	2
Atelectasis	3		
CD3	3		
Pneumonia	2		
Aspiration pneumonia	1	Ileus	2
CD4	4		
Pneumonia	1		
Aspiration pneumonia	2	Ileus	1
Respiratory failure	1		
CD5	2		
Aspiration pneumonia	1		
Respiratory failure	1		

*CD* Clavien-Dindo classification

Table 2 compares perioperative characteristics between the PC and non-PC groups. Age was greater (80 years vs 71 years, *p* < 0.001), frequency of chronic obstructive pulmonary distress was higher (9.3% vs 3.2%, *p* = 0.029), ASA-PS was poorer (*p* < 0.001), the proportion of underweight was higher (42.6% vs 13.4%, *p* < 0.001), frequency of open surgery was greater (24.1% vs 9.3%, *p* < 0.001), and volume of blood loss was greater (40 mL vs 22 mL, *p* < 0.001) in the PC group than in the non-PC group. Other factors including sex, comorbidities, preoperative treatment, clinical TNM status, tumor location, operation time, and combined resection of adjacent structures were similar between groups. Hospital stay was significantly longer in the PC group (21 days) than in the non-PC group (13 days, *p* < 0.001).

**Table 2:** Comparison of clinical and surgical characteristics between pulmonary and non-pulmonary complication groups

	Pulmonary complication group (*n* = 54)	Non-pulmonary complication group (*n* = 3925)	*p* value
Sex			0.057
Female	18 (33.3)	1765 (45.0)	
Male	36 (66.7)	2160 (55.0)	
Age, years (range)	80 (50–94)	71 (23–101)	< 0.001
Comorbidities			0.209
No	17 (31.5)	1593 (40.6)	
Yes	37 (68.5)	2332 (59.4)	
Chronic obstructive pulmonary distress			0.029
No	49 (90.7)	3801 (96.8)	
Yes	5 (9.3)	124 (3.2)	
Preoperative treatment			1.000
No	50 (92.6)	3625 (92.4)	
Yes	4 (7.4)	300 (7.6)	
American Society of Anesthesiologists performance status			< 0.001
1	3 (5.6)	994 (25.3)	
2	29 (53.7)	2539 (64.7)	
3	22 (40.7)	392 (10.0)	
Body mass index, kg/m^2^			< 0.001
Normal weight (18.5 < , < 30)	30 (55.5)	3254 (82.9)	
Overweight (≥ 30)	1 (1.9)	144 (3.6)	
Underweight (≤ 18.5)	23 (42.6)	527 (13.4)	
Clinical T status			0.376
1	8 (14.8)	507 (12.9)	
2	9 (16.7)	885 (22.5)	
3	21 (38.9)	1708 (43.5)	
4	16 (29.6)	825 (21.0)	
Clinical N status			0.408
Negative	34 (63.0)	2236 (57.0)	
Positive	20 (37.0)	1689 (43.0)	
Clinical M status			0.138
Negative	44 (81.5)	3462 (88.2)	
Positive	10 (18.5)	463 (11.8)	
Tumor location			0.485
Colon	46 (85.2)	3151 (80.3)	
Rectum	8 (14.8)	774 (19.7)	
Approach			0.001
Laparoscopic surgery	41 (75.9)	3545 (90.3)	
Open surgery	13 (24.1)	380 (9.3)	
Operation time, min (range)	232 (107–584)	227 (145–871)	0.121
Estimated blood loss, mL (range)	40 (0–2640)	22 (0–5983)	< 0.001
Combined resection of adjacent structures			0.592
No	49 (90.7)	3640 (92.7)	
Yes	5 (9.3)	285 (7.3)	
Hospital stay, days (range)	21 (3–155)	13 (1–207)	< 0.001

Data are presented as number of patients (percentage) or median (range)

Table 3 shows the predictive ability of clinical factors for PC. Univariate analysis revealed greater age, underweight chronic obstructive pulmonary distress, poor ASA-PS, surgical approach, clinical T status, and blood loss as factors significantly associated with PC. Multivariate analysis revealed male sex (odds ratio (OR) 2.165, 95% confidence interval (CI) 1.176–3.986; *p* = 0.013), greater age (OR 3.180, 95%CI 1.798–5.624; *p* < 0.001), underweight (OR 3.961, 95%CI 2.210–7.100; *p* < 0.001), and poorer ASA-PS (OR 3.828, 95%CI 2.144–6.834; *p* < 0.001) as independent predictors of PC.

**Table 3:** Uni- and multivariate analyses predicting postoperative pulmonary complications

	Univariate analysis			Multivariate analysis		
	Odds ratio	95%CI	*p* value	Odds ratio	95%CI	*p* value
Sex			0.090			0.013
Female	1			1		
Male	1.634	0.924–2.887		2.165	1.176–3.986	
Age			< 0.001			< 0.001
< 80 years	1			1		
≥ 80 years	4.319	2.511–7.246		3.180	1.798–5.624	
Body mass index, kg/m^2^						
Normal weight (18.5 < , < 30)	1			1		
Overweight (≥ 30)	0.495	0.068–3.607	0.488	0.799	0.106–6.033	0.828
Underweight (≤ 18.5)	4.783	2.768–8.267	< 0.001	3.961	2.210–7.100	< 0.001
American Society of Anesthesiologists performance status			< 0.001			< 0.001
< 3	1			1		
3	6.196	3.565–10.769		3.828	2.144–6.834	
Comorbidity			0.178			
No	1					
Yes	1.486	0.834–2.649				
Chronic obstructive pulmonary distress			0.017			0.306
No	1			1		
Yes	3.127	1.225–7.986		1.674	0.623–4.502	
Tumor location			0.370			
Colon	1					
Rectum	0.708	0.332–1.506				
Preoperative treatment			0.948			
No	1					
Yes	0.966	0.346–2.694				
Surgical approach			< 0.001			0.356
Laparoscopic surgery	1			1		
Open surgery	2.958	1571–5.569		1.390	0.690–2.803	
Combined resection of adjacent structures			0.575			
No	1					
Yes	1.303	0.515–3.296				
Clinical T status			0.064			0.329
T1–3	1			1		
T4	1.726	0.967–3.081		1.359	0.733–2.519	
Clinical N status			0.249			
Negative	1					
Positive	0.718	0.409–1.260				
Clinical M status			0.133			
Negative	1					
Positive	1.699	0.849–3.399				
Operation time, min			0.684			
< 230	1					
≥ 230	1.117	0.653–1.913				
Estimated blood loss, mL			0.007			0.092
< 40	1			1		
≥ 40	2.132	1.223–3.718		1.684	0.918–3.089	

*CI* confidence interval

## Discussion

The present multicenter study examined risk factors for the development of PC in patients after surgery for colorectal cancer. Patient age was greater, chronic obstructive pulmonary distress was more common, ASA-PS was poorer, BMI was lower, open surgery was more frequent, and volume of blood loss was more in the PC group. Multivariate analysis revealed male sex, high age, and poor ASA- PS as independent predictors for PC.

According to previous reports, the frequency of PC after abdominal surgery is 2–30% [1, 4, 5, 12, 14]. Abd El et al. used the multicenter database to examine 50,150 patients who underwent minimally invasive surgery for colorectal cancer between 2012 and 2017, identifying PC in 637 patients (1.3%) [12]. The incidence of PC in our multicenter database was relatively low, at 1.5%. In addition, approximately 20% of patients with PC die within 30 days, compared to 0.2–3% of patients without PC, representing a very high mortality rate [1]. In the present study, 1.5% of patients developed PC and 3.7% of those who developed PC died within 30 days. One reason for this low mortality rate may be that this study used relatively recent data, with improved perioperative management, a higher frequency of minimally invasive procedures (90.1%), and the active introduction of routine perioperative rehabilitation at all centers. In addition, 30-day mortality may have been lower because the facilities in which PC occurred in this study were equipped with intensive care units staffed by dedicated intensivists, who may have been able to achieve patient recovery using intensive care after complication onset. Of the 54 patients who developed PC, 10 (18.5%) developed intestinal obstruction and 8 (14.8%) experienced delirium. Vomiting and subsequent aspiration due to bowel obstruction after abdominal surgery is a major cause of pneumonia. Aspiration pneumonia is a life-threatening complication with a mortality rate of approximately 27% among high-risk patients [15]. Careful postoperative management of abdominal symptoms is important, as it can be prevented by immediate action, such as insertion of a nasogastric tube during vomiting [15].

Delirium is associated with a high risk of infection and an analysis of patients undergoing planned coronary artery bypass grafting found that patients who experienced delirium had infections significantly more frequently (22%) than those who did not (7.4%, *p* = 0.0037) [16]. Pneumonia and surgical site infection at the sternum were the most common. Delirium is usually reversible but is associated with longer hospitalization, worse prognosis, and increased mortality [17]. PC and delirium may be caused by decreased activities of daily living due to trunk suppression caused by delirium or, conversely, by a cytokine upsurge caused by PC, which may impair optimal oxygenation of the brain.

In the present multivariate analysis of PC, male sex, older age, and poorer ASA-PS were independently associated with poor prognosis.

Motono et al. studied the perioperative outcomes of patients who underwent pneumonectomy, revealing that men were more likely to develop postoperative complications (OR 1.73, 95%CI 1.09–2.75; *p* = 0.01) and postoperative air leakage (OR 1.98, 95%CI 1.03). Schlager et al. reported that patients who underwent left-sided colon cancer surgery developed comorbidities more frequently (OR 1.59, 95%CI 1.10–2.54; *p* = 0.01) than those who underwent right-sided colon cancer surgery (OR 1.98, 95%CI 1.09–2.75; *p* = 0.01) and male sex (OR 1.47, 95%CI 1.21–2.98; *p* = 0.022) as independent risk factors for major complications [18]. One possible explanation is that men generally smoke more than women and have more comorbidities than women, which may significantly impact the occurrence of PC [19].

Bhowmick et al. found that age, smoking, BMI > 25 kg/m^2^, and bi- or unilateral neck dissection were independent predictors of PC after head and neck cancer surgery [20]. Aging is associated with altered immune response, which decreases alveolar macrophage function and increases cell apoptosis during sepsis, leading to more severe infection. In addition, the elderly generally have more fragile and weaker tissues, and blood supply and tissue healing are considered to deteriorate with age, representing a risk factor for infectious complications and supporting our results [21–23].

Laparoscopic surgery has become increasingly popular in recent years, and the pneumoperitoneum and body position applied have been reported to affect PC [7, 24]. The carbon dioxide used for insufflation increases intra-abdominal pressure and decreases both lung compliance and functional residual capacity [7]. The Trendelenburg position, used primarily in the approach to left-sided colorectal cancers, also worsens entry compliance and may cause upper airway edema, ventilatory blood flow imbalance, and increased airway pressure [24]. However, in the present study, the surgical approach was not associated with the occurrence of PC. One reason for this may be the fact that this study investigated relatively recent cases and that the time for insufflation and application of the Trendelenburg position were reduced due to improved anesthesia management and shortened operative time with the application of standardized laparoscopic techniques.

Several limitations to this study should be kept in mind. First, this was a retrospective study of a relatively small number of PC. Second, this study was based on a multicenter database and lacked information on factors that might be associated with the development of PC, such as smoking history, anesthesia method, and postoperative rehabilitation. A larger study with more information is therefore desirable. Third, it is possible that pulmonary complications increase due to postoperative infection. In this study, we sorted pulmonary complications by multicenter database and no infectious complications were found. However, this may be due to an error or omission.

Despite these limitations, our study revealed that male sex, greater age, and poorer ASA-PS are associated with greater risk of PC, and that pre- and postoperative rehabilitation and pneumonia control measures should be implemented for patients at high risk of PC.

## Supplementary Information

Below is the link to the electronic supplementary material.

Supplementary file1 (DOCX 34 KB)

## Data Availability

The datasets generated and/or analyzed during the current study are available from the corresponding author on reasonable request.

## Declarations

Research involving human participants/informed consent

## References

[1] Miskovic A, Lumb AB (2017) Postoperative pulmonary complications. Br J Anaesth 118(3):317–334 28186222 10.1093/bja/aex002

[2] Haines KJ, Skinner EH, Berney S (2013) Association of postoperative pulmonary complications with delayed mobilisation following major abdominal surgery: an observational cohort study. Physiotherapy 99(2):119–125 23219632 10.1016/j.physio.2012.05.013

[3] Zogg CK, Najjar P, Diaz AJ, Zogg DL, Tsai TC, Rose JA Jr et al (2016) Rethinking priorities: cost of complications after elective colectomy. Ann Surg 264(2):312–322 26501705 10.1097/SLA.0000000000001511

[4] Abbott TEF, Fowler AJ, Pelosi P, Gama de Abreu M, Møller AM, Canet J et al (2018) A systematic review and consensus definitions for standardised end-points in perioperative medicine: Pulmonary complications. Br J Anaesth 120(5):1066–1079 29661384 10.1016/j.bja.2018.02.007

[5] Kirmeier E, Eriksson LI, Lewald H, Jonsson Fagerlund M, Hoeft A, Hollmann M et al (2019) Post-anaesthesia pulmonary complications after use of muscle relaxants (POPULAR): a multicentre, prospective observational study. Lancet Respir Med 7(2):129–140 30224322 10.1016/S2213-2600(18)30294-7

[6] Fujii S, Akagi T, Inomata M, Katayama H, Mizusawa J, Ota M et al (2019) Transitional impact of short- and long-term outcomes of a randomized controlled trial to evaluate laparoscopic versus open surgery for colorectal cancer from Japan Clinical Oncology Group Study JCOG0404. Ann Gastroenterol Surg 3(3):301–309 31131359 10.1002/ags3.12245 PMC6524094

[7] Rauh R, Hemmerling TM, Rist M, Jacobi KE (2001) Influence of pneumoperitoneum and patient positioning on respiratory system compliance. J Clin Anesth 13(5):361–365 11498317 10.1016/s0952-8180(01)00286-0

[8] Awad H, Walker CM, Shaikh M, Dimitrova GT, Abaza R, O’Hara J (2012) Anesthetic considerations for robotic prostatectomy: a review of the literature. J Clin Anesth 24(6):494–504 22986320 10.1016/j.jclinane.2012.03.003

[9] Girrbach F, Petroff D, Schulz S, Hempel G, Lange M, Klotz C et al (2020) Individualised positive end-expiratory pressure guided by electrical impedance tomography for robot-assisted laparoscopic radical prostatectomy: a prospective, randomised controlled clinical trial. Br J Anaesth 125(3):373–382 32665059 10.1016/j.bja.2020.05.041

[10] Zhang X, Deng C, Wan Q, Zhao R, Han L, Wang X (2022) Impact of sarcopenia on postoperative pulmonary complications after gastric cancer surgery: a retrospective cohort study. Front Surg 9:1013665 36684364 10.3389/fsurg.2022.1013665 PMC9852346

[11] Zhou L, Li Y, Ni Y, Liu C (2023) Analysis of postoperative pulmonary complications after gastrectomy for gastric cancer: development and validation of a nomogram. Front Surg 10:1308591 38186389 10.3389/fsurg.2023.1308591 PMC10768169

[12] Abd El Aziz MA, Perry WR, Grass F, Mathis KL, Larson DW, Mandrekar J et al (2020) Predicting primary postoperative pulmonary complications in patients undergoing minimally invasive surgery for colorectal cancer. Updates Surg 72(4):977–983 33001373 10.1007/s13304-020-00892-6

[13] Chai J, Sang A, Tan M, Long B, Chen L (2021) Identification of the risk factors of postoperative pulmonary complications in elderly patients undergoing elective colorectal surgery. Am Surg 87(5):777–783 33174436 10.1177/0003134820950304

[14] Schiphorst AH, Verweij NM, Pronk A, Borel Rinkes IH, Hamaker ME (2015) Non-surgical complications after laparoscopic and open surgery for colorectal cancer - a systematic review of randomised controlled trials. Eur J Surg Oncol 41(9):1118–1127 25980746 10.1016/j.ejso.2015.04.007

[15] Studer P, Räber G, Ott D, Candinas D, Schnüriger B (2016) Risk factors for fatal outcome in surgical patients with postoperative aspiration pneumonia. Int J Surg 27:21–25 26804349 10.1016/j.ijsu.2016.01.043

[16] Zukowska A, Kaczmarczyk M, Listewnik M, Zukowski M (2023) The association of infection with delirium in the post-operative period after elective CABG surgery. J Clin Med 12(14):4736 37510851 10.3390/jcm12144736 PMC10380657

[17] Rose L, Burry L, Agar M, Blackwood B, Campbell NL, Clarke M et al (2021) A core outcome set for studies evaluating interventions to prevent and/or treat delirium for adults requiring an acute care hospital admission: an international key stakeholder informed consensus study. BMC Med 19(1):143 34140006 10.1186/s12916-021-02015-3 PMC8211534

[18] Motono N, Ishikawa M, Iwai S, Iijima Y, Usuda K, Uramoto H (2021) Individualization of risk factors for postoperative complication after lung cancer surgery: a retrospective study. BMC Surg 21(1):311 34261455 10.1186/s12893-021-01305-0 PMC8278712

[19] Schlager L, Monschein M, Schüller J, Bergmann M, Krall C, Razek P et al (2023) The predictive value of comorbidities on postoperative complication rates and overall survival in left-sided oncological colorectal resections: a multicentre cohort study. Int J Surg 109(12):4113–4118 37800585 10.1097/JS9.0000000000000734 PMC10720865

[20] Bhowmick RS, Sarkar A, Ghosh S, Gope S, Chakraborty R (2024) Postoperative pulmonary complication as an emerging complication in major head and neck cancer surgery: a retrospective study. Natl J Maxillofac Surg 14(3):471–476 10.4103/njms.njms_399_21 PMC10806327 38273923

[21] Fung M, Jacobsen E, Freedman A, Prestes D, Farmakiotis D, Gu X et al (2019) Increased risk of infectious complications in older patients with indolent non-hodgkin lymphoma exposed to bendamustine. Clin Infect Dis: Official Publ Infect Dis Soc Am 68(2):247–255 10.1093/cid/ciy458 PMC6321852 29800121

[22] Pham C, Kuten SA, Knight RJ, Nguyen DT, Graviss EA, Gaber AO (2020) Assessment of infectious complications in elderly kidney transplant recipients receiving induction with anti-thymocyte globulin vs basiliximab. Transp Infect Dis: Official J Transp Soc 22(3):e13257 10.1111/tid.13257 32031729

[23] Ittisanyakorn M, Ruchichanantakul S, Vanichkulbodee A, Sri-On J (2019) Prevalence and factors associated with one-year mortality of infectious diseases among elderly emergency department patients in a middle-income country. BMC Infect Dis 19(1):662 31345168 10.1186/s12879-019-4301-z PMC6659312

[24] Yu J, Lee Y, Park JY, Hwang JH, Kim YK (2021) Diaphragm thickening fraction as a prognostic imaging marker for postoperative pulmonary complications in robot-assisted laparoscopic prostatectomy requiring the Trendelenburg position and pneumoperitoneum. Dis Markers 2021:9931690 34257750 10.1155/2021/9931690 PMC8245222

